# Problems of variable biomarker evaluation in stratified medicine research—A case study of ERCC1 in non-small-cell lung cancer

**DOI:** 10.1016/j.lungcan.2015.11.017

**Published:** 2016-02

**Authors:** Kinga Malottki, Sanjay Popat, Jonathan J. Deeks, Richard D. Riley, Andrew G. Nicholson, Lucinda Billingham

**Affiliations:** a Cancer Research UK Clinical Trials Unit (CRCTU), MRC Midland Hub for Trials Methodology Research, Institute of Cancer and Genomic Sciences, University of Birmingham, United Kingdom; b Department of Medicine, Royal Marsden Hospital, London SW3 6JJ, United Kingdom; c Institute of Applied Health Research, University of Birmingham, United Kingdom; d Research Institute for Primary Care and Health Sciences, Keele University, United Kingdom; e Department of Histopathology, Royal Brompton and Harefield NHS Foundation Trust and National heart and Lung Institute, Imperial College, London, United Kingdom

**Keywords:** ERCC1, excision repair cross-complementation group 1, FISH, fluorescence in situ hybridization, IHC, immunohistochemistry, NER, nucleotide excision repair, NR, not reported, NSCLC, non-small-cell lung cancer, PD-L1, programmed-death ligand 1, RTqPCR, reverse transcriptase quantitative polymerase chain reactio, ERCC1 protein, Human, Biological markers, Drug therapy, Antineoplastic therapy, Carcinoma, Non-small-cell lung, Clinical trials as topic

## Abstract

**Objectives:**

Consistency of procedures for the evaluation of a predictive biomarker (including sample collection, processing, assay and scoring system) based on adequate evidence is necessary to implement research findings in clinical practice. As a case study we evaluated how a particular predictive biomarker, ERCC1, was assessed in research on platinum-based chemotherapy in non-small-cell lung cancer and what motivated the choice of procedure.

**Materials and methods:**

A systematic review of studies completed since 2007 and ongoing was undertaken. Questionnaires on details of ERCC1 evaluation procedures and the rationale for their choice were sent to contacts of identified studies.

**Results:**

Thirty-three studies of platinum-based chemotherapy in non-small-cell lung cancer using ERCC1 were identified. A reply to the questionnaire was received for 16 studies. Procedures for ERCC1 evaluation varied substantially and included reverse transcriptase quantitative polymerase chain reaction (nine studies), immunohistochemistry (five studies) and other methods (multiple methods–two studies, NER polymorphism–one study). In five studies ERCC1 use was planned, but not undertaken. In nine data was insufficient to identify the procedure. For each assay there was variation across studies in the details of the laboratory techniques, scoring systems and methods for obtaining samples.

**Conclusions:**

We found large variation across studies in ERCC1 evaluation procedures. This will limit the future comparability of results between these different studies. To enable evidence-based clinical practice, consensus is needed on a validated procedure to assess a predictive biomarker in the early phase of research. We believe that ERCC1 is not untypical of biomarkers being investigated for stratified medicine.

## Highlights

• This was a case study of variation in methods of predictive biomarker assessment.
• 33 recent studies of platinum-based chemotherapy in NSCLC using ERCC1 were found.
• Procedures for ERCC1 evaluation varied substantially.
• This will limit comparability of results and their implementation in practice.
• Consensus is needed on a validated procedure to assess predictive biomarkers.

## 1 Introduction

Lung cancer is one of the leading causes of cancer mortality globally [1], [2], [3]. The majority of patients have non-small-cell lung cancer (NSCLC) histology [3], [4]. Prognosis in these patients is generally poor [2], [4], with a five year survival of about 5% for advanced NSCLC and about 15% irrespective of stage [3]. In spite of development of new, targeted treatments, platinum-based chemotherapy remains a major part of NSCLC care [2], [4], [5], [6].

The effectiveness of platinum-based chemotherapy is however limited [1], [7], with resistance to treatment resulting in little or no benefit and potentially unnecessary toxicity in some patients [8]. In a significant number of patients, identification of biomarkers predictive of resistance to platinum-based chemotherapy could potentially result in avoiding unnecessary treatment, as well as better allocation of healthcare resources. Expression of excision repair cross-complementation group 1 (ERCC1) gene has been suggested as a biomarker potentially relevant to prediction of response to platinum-based chemotherapy [2].

The use of predictive biomarkers is becoming more common. The accuracy and replicability of the procedures used to evaluate these biomarkers (including sample collection, processing, assay, scoring system and threshold) are therefore crucial. The use of standardised procedures is important to facilitate combination of results of multiple studies in a meta-analysis and implementation of their findings in clinical practice. There are however reasons to believe that in practice there may be little consistency in these procedures. A review of published papers investigating ERCC1 expression to predict response to platinum-based chemotherapy in lung cancer found that there was large variability in the assays used [2]. This review was published in 2011, thus including relatively early ERCC1 evaluations. There was a possibility that more recent research practice has become more harmonised.

ERCC1 was also chosen as a case study, as the research investigating it as a potential predictive biomarker was relatively recent and therefore likely to illustrate current practice. An interesting development was that it was suggested that currently there may be no laboratory procedure capable of identifying the ERCC1 isoform that may be responsible for resistance to cisplatin [7].

The aim of this systematic review undertaken in 2013 and subsequent questionnaire was to investigate the consistency of methods for evaluation of ERCC1 as a biomarker predictive of response to platinum-based chemotherapy in ongoing or completed since 2007 studies in NSCLC, and to investigate the rationale for choice of a specific method. This project sets out to provide a case study of current research practice, from which lessons can be learned that may apply to a wider context of predictive biomarker research.

## 2 Materials and methods

Searches for studies completed since 2007 and ongoing were undertaken on 26 March 2013 in ClinicalTrials.gov, WHO and the Controlled-Trials databases. Search terms were based on the patient population (NSCLC), the biomarker (ERCC1) and treatment (platinum-based chemotherapy). The full search strategies are available in the online supplement.

Studies meeting the following criteria were included:

• Population: patients with NSCLC (any stage).
• Intervention: at least one of the study arms included platinum-based chemotherapy.
• Biomarker assay: any assay measuring ERCC1 expression or nucleotide excision repair (NER) gene expression in tumour tissue or blood.
• Outcome: any.
• Study: any ongoing study or completed/terminated after 1st January 2007.

Titles of studies were screened by two independent reviewers (KM and LB) and those clearly not meeting the inclusion criteria were excluded. For the remaining studies full records obtained from databases of ongoing trials were considered for inclusion by two independent reviewers (KM and LB). Studies were included if they met all inclusion criteria. Studies only specifying the intervention as chemotherapy or systemic therapy were also included if all the remaining criteria were met. Disagreements between reviewers were resolved by discussion and in two cases by seeking further information on the studies in internet searches.

For all included studies, information was extracted from the databases on: study phase, design, planned sample size, status (ongoing, completed, terminated or withdrawn), start and planned end date, primary outcome, patient inclusion criteria, intervention, ERCC1 evaluation, location, sponsor and contact details.

A questionnaire asking about the details of ERCC1 evaluation procedures and reasons for their choice was prepared in collaboration with clinical and pathology experts and sent to contacts for each included study, the sponsor or for published studies the corresponding author (whichever was available). The questionnaire was sent on 5th August 2013 and if no reply was received, again on 28th January 2014. For completed studies searches for publications were also undertaken.

Data obtained from databases of ongoing trials and replies received were summarised using descriptive analysis. No additional information was obtained through searches for published studies.

## 3 Results

### 3.1 Details of studies included in the systematic review

**Fig. 1:**
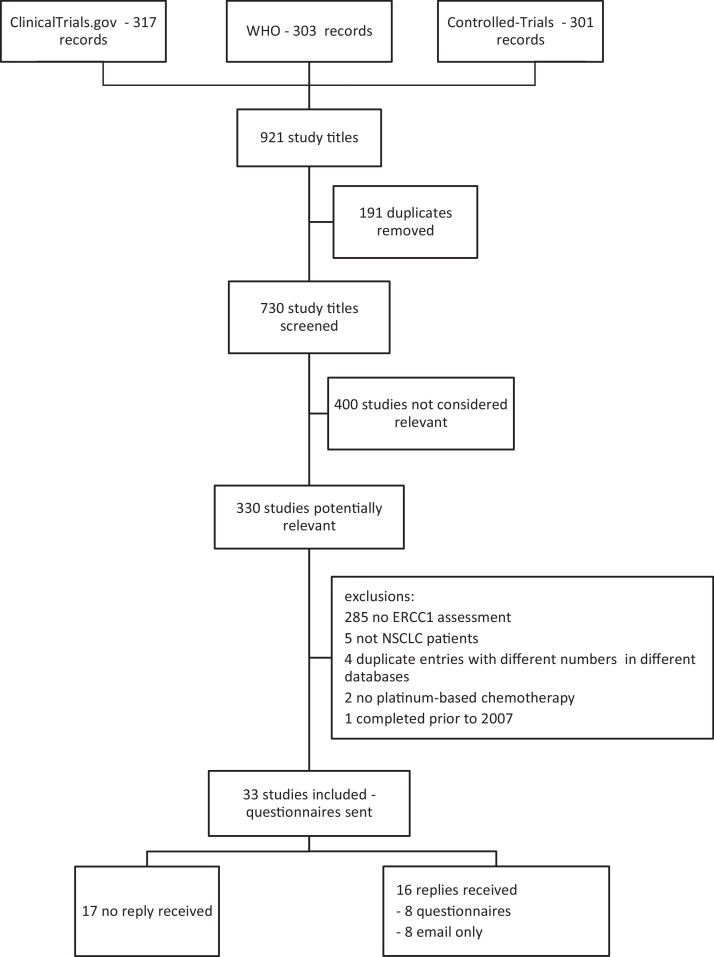
Flow diagram outlining the results of searches in ongoing trials databases, review of studies and replies received for the questionnaire.

The searches identified 730 unique records in databases of clinical trials. The review process is presented in detail in Fig. 1, leading to 33 studies being included in the study.

**Fig. 2:**
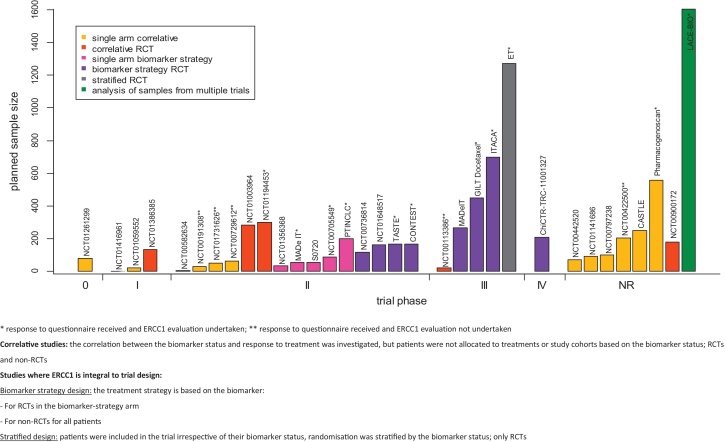
Planned trial sample size and design with respect to ERCC1 by trial phase in identified studies.

Eighteen of the included studies were ongoing, eight completed, two terminated early and one withdrawn prior to enrolment. The status of four studies was unknown. Nine of the included studies were conducted in Asia, eight in Europe, 13 in North America, one included locations in Europe and North America and for two the location was not reported. The phase and size of studies together with design is shown in Fig. 2.

There were two key types of study design (see Fig. 2 caption for details). In 19 studies ERCC1 was not an integral part of the study design, but a correlation between the biomarker status and treatment outcome was investigated (correlative studies). The remainder used ERCC1 as an integral part of the design: thirteen used ERCC1 alone or in combination with other biomarkers to determine treatment strategy (biomarker strategy design) and one study used ERCC1 to stratify randomisation.

As expected, single arm correlative studies were most frequently early phase studies (phase 0, I and II). Nine of 15 phase II studies reported testing a strategy that was based on ERCC1 and in some cases also included other biomarkers. Phase III trials included one correlative RCT, one RCT stratified by ERCC1 and three RCTs using ERCC1 to select a treatment strategy. The phase IV study was a biomarker-based strategy RCT.

Detailed characteristics of included studies are reported in the online Supplement.

### 3.2 ERCC1 Information on all included studies

**Fig. 3:**
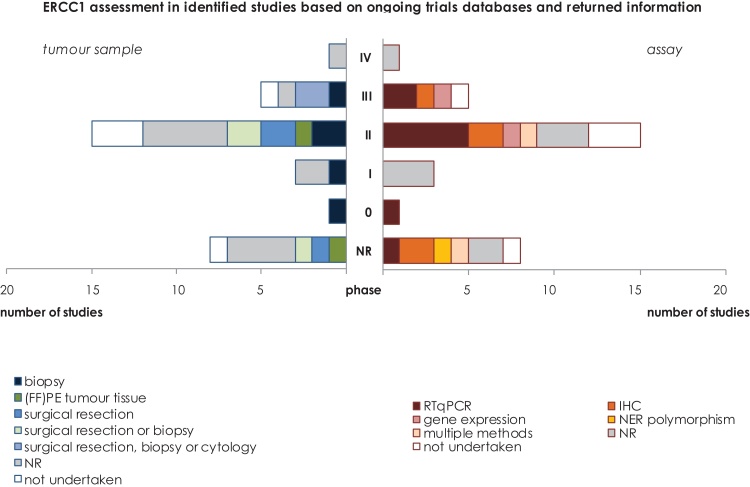
ERCC1 evaluation in identified studies.

The procedures for evaluation of ERCC1 varied across studies (Fig. 3). Data was available in sufficient detail to enable the identification of the laboratory procedure used in 24 of the 33 studies. Of these, reverse transcriptase quantitative polymerase chain reaction (RTqPCR) was used in nine (38%) and immunohistochemistry (IHC) in five (21%) studies. Two studies reported the use of multiple methods. In one immunofluorescence-based automated quantitative analysis for in situ expression was used as the primary assay and if additional samples were available, RT-PCR, RTqPCR, polymorphism analysis and tissue microarray analysis of genes associated with DNA synthesis, damage repair, and drug efficacy were also undertaken. In another study both fluorescence in situ hybridization (FISH) and IHC were used. One study investigated NER polymorphism. In five studies, although initially planned, no ERCC1 evaluation was undertaken.

The type of specimen used also varied, as shown in Fig. 3, with biopsy being the most frequent.

It appears that irrespective of the study phase there was variation in the laboratory procedures used for evaluation of ERCC1 status. There was no relationship between the year of study initiation and laboratory procedure (data in Supplementary figure).

### 3.3 Additional Information on ERCC1 evaluation obtained from the questionnaire

A reply to the questionnaire was received for 16 studies (shown in Fig. 2). Five of these did not undertake ERCC1 evaluation, although initially planned (reasons included: early study termination, insufficient samples, lack of funding).

In the 11 studies that evaluated ERCC1, it was prospective (prior to patients receiving treatment) in all eight studies using the biomarker to identify treatment strategy or stratify randomisation. Three studies were correlative and all of these evaluated ERCC1 retrospectively and blind to patient outcome. In studies assessing ERCC1 prospectively the time needed for results to be returned to the treating physician varied between a minimum of one to two days to 14 days. It was however usually not indicated if this time was measured from patient enrolment or receipt of sample by the laboratory.

Nine of the replies reported the site of ERCC1 evaluation: in seven it was a central laboratory and in two an individual hospital.

Of the five studies where ERCC1 was evaluated with IHC, the monoclonal 8F1 antibody clone was used in three (in 1:300 dilution in two and not reported in one), the ZSGB-Bio, China antibody clone in one (in 1:50 dilution) and it was not reported in one. Ancillary methods were reported for two of these studies and were: automated ICH stainer in one study and exposing samples to 10 mM citrate buffer (pH 6.0) and then heating for 30 min in a water bath in the other.

To obtain an IHC expression score four studies used the H-score and one study used the Allred Quick Score. The thresholds for classifying patients as positive were:

• H-score 1 and above in two studies,
• median H-score in one study (retrospective analysis),
• Allred Quick Score 6 and above.

In six studies which used RTqPCR it appears that different sets of primers were used, although this could not be established with certainty (details shown in Table 1). β-actin on its own was used as the reference gene in four studies. In one study β-actin was used together with PGK and in one study 18SrRNA was used. Only two studies reported the method used to calculate the quantity of ERCC1 RNA and it was the ΔΔ*CT* method. The thresholds for classifying patients as positive were:

• median in three studies,
• ratio of ERCC1 to reference gene transcripts of 1.7 in one study,
• 8.7 (no further details provided) in one study,
• in one study the threshold was not clearly reported.

The proportion of patients classed as ERCC1 positive was reported for two studies using RTqPCR and was 0.6 and 0.64.

### 3.4 Rationale for choice of ERCC1 procedures

The rationale for the choice of the procedure varied across studies (shown in Table 2). The reasons provided were experience of the laboratory, published literature, previous research experience (for example pilot study), belief that the method of choice was superior or limitations imposed by the type of the available samples. For one study it was declared that as with current knowledge there are no antibodies that were isoform-specific, there was no rationale for selection of the laboratory procedure.

## 4 Discussion

Application of stratified medicine in the real world requires that the measurement of biomarkers and associated classification algorithms used to stratify patients follows a standardised protocol within clinical trials, especially in the later phases. This ensures that the evidence-base behind the stratified treatment is consistent and valid, so that evidence-based decisions can be made about whether a particular marker can be utilised in practice and, if so, how. We have undertaken to research this issue via a specific case-study and our aim was to investigate whether laboratory procedures used for ERCC1 evaluation have become more standardised since a meta-analysis published in 2011 found large variation [2].

There were 33 studies that met our inclusion criteria, ranging from phase 0 to phase IV. Fifteen of the studies used ERCC1 as an integral part of their design: either to allocate treatment or to stratify patients.

Our findings suggest that there was still large variation in both the laboratory procedures and the tumour specimens used for ERCC1 evaluation. Although they attempt to evaluate the same biomarker, some small studies suggest that classifying patients as ERCC1 positive and negative based on either RTqPCR or IHC can lead to relatively large discrepancies [9], [10]*.* For example, one study investigating samples from 91 patients found that there was a statistically significant correlation between the ERCC1 mRNA and protein expression levels. However when thresholds for classifying patients as positive and negative were used, 33% of tumours ERCC1 negative by RTqPCR were IHC positive and 32% IHC-negative tumors were classed as ERCC1 positive using RTqPCR [11]. These findings suggest that both techniques may not be interchangeable.

In our review even when the same assay was used, details of the laboratory procedures and scoring systems appeared to vary. This could further decrease the comparability of results between studies.

In the questionnaire, three out of five studies using IHC reported using the 8F1 antibody clone (Nomarkers). In two of the three studies using the 8F1 clone, the dilution was reported and it was the same. The use of the same antibody clone is crucial, as different clones for the same antigen bind to different epitopes and can therefore have different sensitivities and specificities [12], [13].

There was large variation in the techniques used for RTqPCR, especially in terms of the primers used, which can have a substantial impact on the results obtained using this method [14]. Five of the six studies used β-actin as the reference standard (in one case together with another gene). The choice of a reference standard can be challenging and several publications have suggested that levels of β-actin expression can vary and may not be a good reference standard [15], [16]. The thresholds chosen in individual studies also varied and, interestingly, three studies chose the median value obtained within the study. This seems to imply an underlying assumption that half of the patients in these studies are resistant to platinum-based chemotherapy due to ERCC1 overexpression, however no reason for this assumption was provided.

Apart from using different assays, the methods of tumour sample collection also varied. A small study using IHC for ERCC1 assessment suggested that there might be a discrepancy in classifying patients' ERCC1 expression levels depending on whether tumour tissue was obtained using biopsy or surgical resection [17]. Another study found discrepant results depending on whether a tumour sample was obtained from the primary tumour or a metastatic site [18].

Where reported, the proportion of patients classed as ERCC1 positive ranged from 0.25 to 0.78. This would further suggest that procedures and criteria used in different studies for classifying ERCC1 expression levels do not produce comparable results. It is however possible that this variation is largely due to chance or, for example, prognostic properties of ERCC1.

On undertaking this review and questionnaire it was hypothesised that the highest variation in the assays for ERCC1 evaluation should be seen in early phase trials and higher levels of standardisation were expected for later phase studies. This was however not observed. There was relatively large variation in the methods chosen for ERCC1 evaluation in phase II and III trials. There was also no evidence of a trend suggesting certain methods of ERCC1 evaluation became more popular at a particular time (for example due to publication of research suggesting one method could be superior).

With regards to the rationale for the choice of a particular method, it was often motivated by experience of either the laboratory or the researchers involved, although for three studies published literature was also referred to.

As recent research suggests, there may be no ERCC1 assay capable of identifying a subgroup of patients more likely to benefit from platinum-based chemotherapy. When tumour tissue samples from the same patients originally enrolled in the IALT trial were re-evaluated using exactly the same IHC procedures, 36% of patients classed as ERCC1 positive on one occasion were classed as negative on another.[7]. This raises the issue of unnecessarily enrolling patients in trials where ERCC1 is, or was integral to trial design. This potentially resulted in suboptimal treatment of these patients and a suboptimal allocation of resources.

This systematic review is based on a relatively small sample of studies and detailed information (from the questionnaire) was limited to 16. The objective here was however not of quantitative nature, but mainly to collect information on an example of large discrepancies in evaluation of a potential predictive biomarker. There is no reason to believe that this example is not representative of at least some stratified medicine research, and the conclusions are unlikely to change if further studies had been obtained (indeed, heterogeneity in laboratory methods would likely increase).

From the perspective of reviewing evidence and implementing biomarkers in clinical practice, it would be ideal if there was one valid laboratory procedure used for biomarker evaluation in all studies. However in practice this is unlikely, as the technology in this field is rapidly developing. New laboratory procedures are being developed, which can offer more accurate biomarker evaluation, as well as cost and time savings. It is therefore not surprising that these are implemented in studies (although our review did not identify an effect of dissemination of new technologies on the choice of the laboratory procedures). Some of the variability may also be due to the competition between different research groups and differences in opinion on the suitability of a given assay. This presents a challenge for implementing findings of studies using different procedures in clinical practice, especially that, as some research on ERCC1 suggests, the results obtained using different procedures may not be comparable. Therefore there is a need for more research to ensure that procedures used to evaluate the same predictive biomarker actually stratify patients into comparable cohorts.

This example highlights the need for a more structured approach to development and validation of biomarker tests prior to their use in clinical trials. Although it may appear that studies are using the same assay, variation in important details of the laboratory procedures may result in lack of comparability between results of different studies.

It appears that the case of ERCC1 is not isolated. A potentially similar situation has been recently identified in programmed-death ligand 1 (PD-L1) testing in NSCLC, where multiple IHC assays using different antibody clones are under development for four different drugs and there is still uncertainty with regards to how well these assays can predict patient response [19].

## 5 Conclusions

If a biomarker is to be used in clinical studies, especially later phase, ideally its accuracy should be established. There also needs to be consensus on a standardised validated protocol to be followed in clinical trials, which would ensure there is a definitive biomarker evaluation procedure to be used in future clinical practice. Existing international research infrastructure could potentially be utilised to accomplish consensus, however this may not be straightforward due to individual opinions and experience.

## Conflicts of interests

K.M. and S.P. declared no conflicts of interest; J.D. reported institution in receipt of a CRUK project grant; R.R. reported a pending MRC grant and being a Statistics Editor for the BMJ; A.N. provided consultancy for Merck, Boehringer Ingelheim, Pfizer, Novartis and Astra Zeneca, was payed for lectures for Eli Lily and Astra Zeneca and was payed for travel to IASLC Pathology Committee Meetings; L.B. was a member of Eli Lilly Advisory Board and was paid for lectures by Roche.

## Figures and Tables

**Table 1:** Details of RTqPCR used in studies where information was returned.

Study	Primers	Reference gene(s)	Threshold chosen
EUCTR2008-001764-36-IT (ITACA)	Exons-spanning	β-Actin	Median (using ΔΔCT method, value NR)
EUCTR2011-005267-24-IT (CONTEST)	Not known (carried out by external laboratory)	β-Actin	Ratio of ERCC1 to reference gene transcripts: 0.14 (low), 13.4 (high), Cut-off: 1.7
NCT00174629 (GILT Docetaxel)	Designed according to their Ref. Seq in https://www.ncbi.nlm.nih.gov/sites/entrez?db/gene	β-Actin	Median (3.42 using ΔΔCT method)
NCT00705549	“Primers have been previously described in details (Papadaki et al BR J Ca)”–paper could not be identified	β-Actin and PGK	Unclear (“the cut-off was based on the a chart analysis in >800 samples”)
NCT01194453	Primers spanning exons 7–9 of the ENST00000300853 ERCC1 transcript: 5′TCGTCTCCCGGGTGACTG 3′and5′TTCTCTTGATGCGGCGATGAG 3	β-Actin	Median (value NR)
NCT00215930 (MADe IT)	Intron-spanning primers	Housekeeping gene 18SrRNA	Above/below 8.7

**Table 2:** Rationale for the choice of method of ERCC1 assessment in studies for which information was provided.

	Published literature	Previous own research experience	Laboratory experience	Believe method most appropriate	Method suitable for available samples	None	NR
EUCTR2007-007639-17-GB (ET)	✓	✓					
EUCTR2008-001764-36-IT (ITACA)			✓				
EUCTR2011-005267-24-IT (CONTEST)				✓			
NCT00174629 (GILT Docetaxel)			✓				
NCT00705549			✓	✓			
NCT00775385 (TASTE)							✓
NCT01194453					✓		
NCT01294280 (LACE-BIO)	✓						
NCT01781988 (PTINCLC)							✓
NCT00215930 (MADE IT)	✓	✓					
NCT00222404 (Pharmacogenoscan)						✓	
