# Supplementary material for: Problems of variable biomarker evaluation in stratified medicine research—A case study of ERCC1 in non-small-cell lung cancer
Source: Lung Cancer. 2016 Feb;92:1–7. doi: 10.1016/j.lungcan.2015.11.017 (PMC4729317; doi:10.1016/j.lungcan.2015.11.017)
Supplement: Supplementary file 1 [file mmc1.docx]

**Supplementary data**

Search strategies used in ongoing trials databases to identify studies assessing ERCC1 expression are shown below for each database separately.

**ClinicalTrials.gov** (317 hits):

(lung OR NSCLC) AND (customized OR individualized OR tailored OR personalized OR biomarker OR ERCC1OR ERCC OR ERCC-1 OR (excision AND repair) OR pharmacogenomic OR pharmacogenetic) AND (cisplatin OR carboplatin OR platinum OR platin OR chemotherapy)

**WHO** (303 hits):

Lung AND customized OR Lung AND individualized OR Lung AND tailored OR Lung AND personalized OR Lung AND biomarker OR Lung AND ERCC1 OR Lung AND ERCC-1 OR Lung AND ERCC OR Lung AND excision AND repair OR Lung AND pharmacogenomic OR Lung AND pharmacogenetic OR NSCLC AND customized OR NSCLC AND individualized OR NSCLC AND tailored OR NSCLC AND personalized OR NSCLC AND biomarker OR NSCLC AND ERCC1 OR NSCLC AND ERCC-1 OR NSCLC AND ERCC OR NSCLC AND excision AND repair OR NSCLC AND pharmacogenomic OR NSCLC AND pharmacogenetic

(WHO portal does not recognise brackets; operator priority: NOT, AND, OR)

**Controlled-Trials** (301 hits):

(lung OR NSCLC) AND (customized OR individualized OR tailored OR personalized OR biomarker OR ERCC1 OR ERCC OR ERCC-1 OR (excision AND repair) OR pharmacogenomic OR pharmacogenetic)

Figure: Assays for evaluation of ERCC1 expression used in identified studies by date of study initiation

Table: Characteristics of included studies

| Trial ID | Trial status | Reply received | Start - end year | Type and role of chemotherapy; NSCLC stage | Design | Sample size | ERCC1 assessment method* |
| --- | --- | --- | --- | --- | --- | --- | --- |
| ***Phase 0*** | | | | | | | |
| **NCT01261299** | Ongoing | No | 2010 - 2013 | Carboplatin; palliative  Stage IV | uncontrolled, correlative | 80 | quantitative RT-PCR; details NR |
| ***Phase I (including phase I/II)*** | | | | | | | |
| **NCT01059552** | Ongoing | No | 2009 - 2013 | Cisplatin; unclear  Stage IIIa/IIIb | uncontrolled correlative | 22 | NR |
| **NCT01416961** | Withdrawn | No | 2011 - 2011 | Cisplatin; unclear  Stage IIIa/IIIb | uncontrolled, correlative | 0 | NR |
| **NCT01386385** | Ongoing | No | 2011 - 2016 | Carboplatin; only treatment  Stage III | RCT, correlative | 132 | NR |
| ***Phase II (including phase II/III)*** | | | | | | | |
| **EUCTR2011-005267-24-IT** (**CONTEST)** | Ongoing | Yes (questionnaire) | 2012 - 2014 | Cisplatin; neoadjuvant  Stage IIIa | Randomised biomarker - strategy design (using ERCC1, RRM1, EGFR and TS) | 168 | *RTqPCR; ERCC1 RNA level as ratio of ERCC1 gene transcripts to β-actin reference gene transcripts; cut-off is 1.7* |
| **NCT00775385** (**TASTE)** | Ongoing | Yes (email + conference presentation) | 2009 - 2014 | Cisplatin; adjuvant  Stage II/IIIa | Randomised biomarker - strategy design (using ERCC1 and EGFR) | 165 | *IHC; details NR* |
| **NCT00792701** (**S0720)** | Ongoing | No | 2008 - 2016 | Cisplatin; adjuvant  Stage Ia/Ib | uncontrolled biomarker strategy (using ERCC1 and RRM1) | 55 | immunofluorescence-based automated quantitative analysis; if available, additional samples using RT-PCR and RTqPCR, polymorphism analysis for ERCC1 expression at protein level; tissue microarray analysis of genes associated with DNA synthesis, damage repair, and drug efficacy |
| **NCT01003964** | Ongoing | No | 2009 - 2013 | Cisplatin; unclear  Stage IIIb/IV | RCT, correlative | 284 | NR |
| **NCT01194453** | Ongoing | Yes (questionnaire) | 2009 - 2012 | Cisplatin; first-line  Stage IIIb/IV | Randomised, correlative | 300 | *RTqPCR; median value as threshold (value NR);* details NR |
| **NCT01356368** | Ongoing | No | 2010 - 2013 | Cisplatin; first line  Stage IIIb/IV | uncontrolled biomarker strategy (using ERCC1, β-Tubulin and RRM1) | 35 | NR |
| **NCT01731626** | Ongoing | Yes (email) | 2013 | Cisplatin;p neoadjuvant and adjuvant  Stage Ib to IIIb | uncontrolled, correlative | 52 | *Not undertaken* |
| **NCT00705549** | Terminated | Yes (questionnaire) | 2008 - 2011 | Cisplatin; unclear  Stage IIIb/IV | uncontrolled biomarker strategy (using ERCC1, BRCA1 and RRM1) | 88 | *RTqPCR with threshold based on the a chart analysis in >800 samples;* details NR |
| **NCT00191308** | Completed | Yes (email + conference abstract) | 2005 - 2010 | Cisplatin; neoadjuvant  Stage Ib to IIIa | single-arm, correlative | 30 | *not conducted due to insufficient tumor samples and ”lack of scientific value”* |
| **NCT00582634** | Completed | No | 2004 - 2007 | Cisplatin; adjuvant  Stage Ib to IIIa | uncontrolled correlative | 4 | NR |
| **NCT01781988** (**PTINCLC)** | Ongoing | Yes (questionnaire) | 2009 - 2013 | Carboplatin; NR  NR | biomarker-strategy design based on ERCC1, RRM1, TS and β-Tubulin | 200 | *IHC using ZSGB-Bio in China antibody; H-score >1 classed as ERCC1 high* |
| **NCT01648517** | Ongoing | No | 2012 - 2015 | Carboplatin; unclear  Stage IIIb/IV | biomarker-strategy design based on ERCC1 and RRM1 | 162 | mRNA expression; details NR |
| **NCT00736814** | Unknown | No | 2008 - NR | Carboplatin; only treatment  Stage IIIb/IV | biomarker-strategy design based on ERCC1 and RRM1 | 117 | RT-PCR; details NR |
| **NCT00729612** | Unknown | Yes (email) | 2008 - 2010 | Carboplatin; NR  Stage IIIb/IV or recurrent | uncontrolled correlative | 63 | *Not undertaken* |
| **NCT00215930** (**MADe IT)** | Completed | Yes (email + publications) | 2004 - 2009 | Carboplatin; only treatment  Stage IIIb/IV | uncontrolled biomarker strategy (using ERCC1 and RRM1) | 53 | *RTqPCR using ABI prism 7700; Perkin-Elmer, Foster City, CA; threshold: ERCC1 expression above/ below 8.7* |
| ***Phase III*** | | | | | | | |
| **EUCTR2007-007639-17-GB** (**ET)** | Ongoing | Yes (questionnaire) | 2008 - 2014 | Cisplatin; only treatment  Stage IIIb/IV | Randomised stratified (by ERCC1) | 1272 | *IHC using Neomarkers (ThermoFisher) clone 8F1 antibody; threshold: Quick Score 6 and over* |
| **EUCTR2008-001764-36-IT** (**ITACA)** | Ongoing | Yes (email + conference poster) | 2008 - NR | Cisplatin; adjuvant  Stage II/IIIa | Randomised biomarker - strategy design (using ERCC1 and TS) | 700 | *RTqPCR* *using 7900 ABIPRISM; values dichotomized on median value (ΔΔCT method; value NR)* |
| **NCT00113386** | Terminated | Yes (email) | 2005 - 2009 | Cisplatin; neoadjuvant  Stage IIIa | RCT (ERCC1 in secondary correlative analysis) | 19 | *Terminated early due to poor accrual* |
| **NCT00174629** (**GILT Docetaxel)** | Completed | Yes (questionnaire) | 2001 - 2007 | Cisplatin; only treatment  Stage IIIb/IV | biomarker - strategy design (using ERCC1) | 449 | *RTqPCR; threshold using median (ΔΔCT 3.42)* |
| **EUCTR2008-000617-30-DE** (**MADeIT)** | Ongoing | No | 2008 - 2015 | Carboplatin; only treatment  Stage IIIb/IV | biomarker-strategy design based on ERCC1 and RRM1 | 267 | ERCC1 expression at protein level; details NR |
| ***Phase IV*** | | | | | | | |
| **ChiCTR-TRC-11001327** | Ongoing | No | 2010 - 2013 | Cisplatin; NR  Stage IIIb/IV | Randomised biomarker - strategy design, (unclear if ERCC1 used) | 210 | NR |
| ***Phase NR*** | | | | | | | |
| **NCT01294280** (**LACE-BIO)** | Ongoing | Yes (questionnaire) | 2008 - 2013 | Cisplatin; adjuvant  Early stage | analysis of samples from completed trials | 1606 | *IHC using Ab-2, clone 8F1 (Neomarkers) with H-score >1 classed positive* |
| **NCT00900172** | Unknown | No | 2008 - 2009 | Carboplatin; unclear  Stage IIIb/IV | RCT, correlative | 180 | Polymorphisms in ERCC-1 assessed using Taqman assays; details NR |
| **NCT00797238** | Unknown | No | 2007 - 2010 | platinum-based; neoadjuvant  Stage III | uncontrolled, correlative | 100 | NR |
| **NCT00222404** (**Pharmacogenoscan)** | Completed | Yes (questionnaire) | 2005 - 2010 | platinum-based; unclear  Any stage | uncontrolled, correlative | 556 | *IHC; details NR* |
| **NCT01141686** | Completed | No | 2009 - 2009 | platinum-based; unclear  Stage NR | uncontrolled, correlative | 90 | FISH and IHC; details NR |
| **NCT01574300** (**CASTLE)** | Ongoing | No | 2010 - 2017 | Unclear; unclear  Stage IV | uncontrolled, correlative | 250 | NR |
| **NCT00422500** | Completed | Yes (email) | 2003 - 2010 | Unclear; unclear  Stage III to IV | uncontrolled, correlative | 204 | *Not undertaken* |
| **NCT00442520** | Completed | No | 2006 - 2008 | Unclear; unclear  Stage NR | uncontrolled, correlative | 70 | NR (SNPs in ERCC1 gene) |

** information in italics is based on the returned questionnaires and emails received; NR – not reported*
